# Translating and communicating evidence on allergy prevention in children to parents: implementation study protocol

**DOI:** 10.1186/s13690-025-01534-2

**Published:** 2025-02-14

**Authors:** Jonas Lander, Hala Altawil, Christian Apfelbacher, Eva Maria Bitzer, Susanne Brandstetter, Barbara Fillenberg, Marius Hartmann, Christine Holmberg, Julia von Sommoggy, Marie-Luise Dierks

**Affiliations:** 1 Hannover Medical School, Institute for Epidemiology, Social Medicine and Health Systems Research, Hannover, Germany; 2 Institute of Social Medicine and Health System Research, Medical Faculty, Otto Von Guericke University Magdeburg, Magdeburg, Germany; 3 Department of Public Health and Health Education, Freiburg University of Education, Freiburg, Germany; 4 University Children’s Hospital Regensburg (KUNO), Hospital St. Hedwig of the Order of St. John, University of Regensburg, Regensburg, Germany; 5 Chair for Midwifery Science, Johannes Gutenberg University Mainz, Mainz, Germany; 6 Institute for Social Medicine and Epidemiology, Brandenburg Medical School Theodor Fontane, Brandenburg an der Havel, Germany

**Keywords:** Allergy prevention, Health information, Health professionals, Evidence transfer, Implementation study

## Abstract

**Background:**

When seeking advice on allergy prevention in early childhood (Early Childhood Allergy Prevention, ECAP), parents often turn to health and social care providers, such as paediatricians, midwives, and family centres. However, these actors fulfil various, often care-related, roles, and cannot be considered ‘health information providers’ by default. In addition, although the scientific evidence for ECAP is often known by health professionals, it is not actively communicated. In this study protocol, we describe the planned procedure for the development and implementation of a process to communicate ECAP information to parents, with a focus on reaching out to those from migrant communities. Thereby, we also aim to contribute to the understanding of how to design more robust approaches to deliver health information.

**Methods:**

We have chosen the Implementation Research Logic Model as our framework for a multi-stage process. Firstly, we will map regional and local health and social care providers to find potential providers of ECAP information. We will then approach actors from each mapping category for qualitative interviews to assess facilitators and barriers to implementation. Next, we will define actions to ease the implementation process, develop exemplary ECAP information materials for parents, and deliver these to pre-selected health and social care individuals and organizations. Each step will be adapted to meet the needs and preferences of culturally and linguistically diverse populations. Finally, the process will be evaluated for key implementation outcomes (e.g., acceptability, feasibility, effectiveness) by interviewing information providers and surveying information recipients.

**Discussion:**

From a Public Health perspective, studies seem warranted that investigate how evidence from health research may be effectively communicated to the public, rather than merely focusing on, e.g., intervention development. Also, it has often been highlighted that the dissemination of health information needs to better target those who face the greatest difficulties when seeking advice, i.e. individuals/parents who recently migrated. ECAP is a good use case, as scientific evidence is constantly evolving, and the communication of information is hampered by low awareness of high quality sources.

**Table:** 

Text box 1. Contributions to the literature
• The study design should allow for an investigation of how health and social care providers can robustly disseminate health information, through the lens of scientific evidence translation and communication.
• The planned approach will enable the investigation of the so-far largely neglected question of implementation strategies for people to find and use health information
• This study design shows how an implementation science framework can systematically be applied throughout each phase of a public health research project.

## Background

### Early childhood allergy prevention by parents

Preventing allergies in children at an early age is crucial to reducing the burden of this chronic disease on individual health and the healthcare system, as the risk of developing an allergy can be reduced when the immune system is still developing [1]. Expectant and new parents have a crucial role to play, as they need to know when and how to reduce the risk for allergies in their child, for example when and how to introduce different nutrients to a child’s diet. Early childhood allergy prevention (ECAP) is usually implicitly taught to parents alongside more general issues of child health, nutrition and hygiene [2]. As a result, parents are rarely aware of specific prevention practices, especially if there is no particular risk of their child developing an allergy [3, 4]. In addition, they often do not have sufficient knowledge of important details about allergies, especially risk perception [5].

### Health information sources and health literacy in the case of ECAP

Previous research indicates that – although preferences for health information sources differ across population groups, for example due to cultural habits [6] – direct interaction with health and social care providers is a key way for parents to learn about child health [7]. In addition to health care providers (HPs), research on health information for chronic diseases, including allergies, shows that its users also cite other actors as relevant sources. These include for example pharmacists, health insurers, counselling centers, and support/self-help groups [8, 9], and, in the case of parents, child day care centres [7]. However, research suggests that professionals who are in regular contact with parents rarely communicate the topic of ECAP, even when being aware of respective recommendations [2, 10].

The preference for receiving health information through personal interaction with trusted institutions and professionals is in line with the conceptualization of health literacy (HL), of which at least two aspects need to be considered. Firstly, representative studies in Germany have shown that individuals have difficulties in navigating health information, with 82% reporting difficulties in determining trustworthiness [11]. A particular reason for this is the quantity, structure and quality of the information available, especially from digital sources [12]. Secondly, the concept of HL emphasizes organizations’ role and responsibility in enabling informed health decisions [13]. However, existing organizational efforts to ease access to and use of health information in Germany have been widely labelled as inadequate (e.g. [14]). Further, it has been observed that in Germany there is as yet no comprehensive “landscape” of health information, and because of this, on-site/local institutions that do provide such services are rather unknown by the public [12]. As a consequence, it may be unsurprising that with regard to ECAP, we found that parents are largely unaware of readily available and trustworthy sources of ECAP information provided by official institutions [15].

### Situational and organizational impact on access to health/ECAP information

In addition, HL research highlights the impact of social determinants and socio-cultural background on health information practices, needs and preferences, for example Lee et al. (2013) [6]. Schaeffer et al. (2021) point to the statistically significant association between low HL and direct experience of migration [11]. Specific challenges can arise from language barriers and inherited practices and norms; for example when people are not (yet) confident navigating a country’s health system to find information and advice, and thus use general-purpose online search instead. However, previous research on parental health information practices, has emphasized parents’ activities, preferences, etc., rather than how information access and use are influenced by information providers. Examples include research on motivations for seeking information (e.g. in case of concrete disease symptoms) [16], sources of information (e.g. the frequent use of unspecific results in Google) [17], and search behaviour and preferences (e.g. the importance placed on advice from family and friends) [18].

For Germany, Hommes et al. (2021) provide a comprehensive overview of the landscape of supra-regional public health institutions [19]. According to the authors, there is a need to intensify assessments for specific health issues, as there is likely to be considerable variation in the suitability of a particular actor to implement interventions. Focusing on how health and social care institutions^1^ and organizations can support individuals to use health information appropriately also requires understanding which actors can promote HL and what resources they have to do so. From the perspective of strengthening parental HL to make decisions about ECAP, it needs to be understood how information can be provided effectively, as there is no evidence on facilitators and barriers with respect to health and social care providers.

## Methods/design

### Aims and objectives

The planned study is part of the interdisciplinary public health research consortium HELICAP (“Health literacy in early childhood allergy prevention”). HELICAP, funded by the German Research Foundation for a period of six years, assesses ECAP from a public health perspective, for instance regarding how health professionals communicate evidence to parents, and to what extent mothers and fathers behave in a health-literate way during pregnancy and early childhood [20, 21].

In this study protocol, we describe how this planned study will aim to understand how health and social care institutions communicate ECAP information to parents, and what factors facilitate or hinder this process. The specific research objectives are to 1) assess which local and regional health and social care agencies are available to deliver ECAP information, and their respective barriers, facilitators and preferences for implementation (pre-implementation analysis); 2) develop an implementation strategy and test the provision of ECAP information in health and social care settings (implementation planning and implementation) – specified for different groups of parents according to socio-cultural backgrounds; and 3) evaluate implementation outcomes from the perspective of ECAP information providers and recipients (evaluation).

### Study design and conceptual framework

**Fig. 1:**
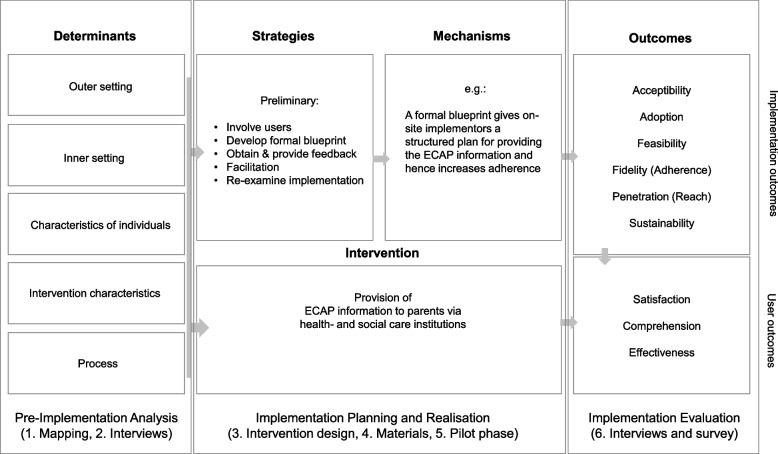
Implementation Research Logic Model adjusted for this study

Overall, the planned study entails six steps related to planning, conducting and evaluating the implementation process, structured according to the Implementation Research Logic Model (IRLM) by Smith, Li and Rafferty (2020) [22]. The IRLM integrates central components of evidence-based implementation research, including implementation determinants (constituted by the Consolidated Framework of Implementation Research, CFIR) [23], implementation strategies [24] and implementation outcomes, and therefore provides crucial guidance throughout the process [25, 26] (Fig. 1).

Besides this theoretical guidance, a panel/board consisting of parents of young children will inform various steps of this research project. This will be carried out according to the principles and steps outlined by one of the major German manuals for participatory health research [27]. To select panel members, we will approach parents who were previously part of a similar board in the project that preceded this study [28]. We plan to have a panel of *n* = 5–7 members to allow for spontaneous unavailability of individual members, given parents’ high involvement in family and work duties. To keep participation for panel members feasible and attractive, we will suggest two meetings and two opportunities for providing written feedback per year. We will therefore define relevant feedback phases, e.g. to develop interview contents, decide on the selection of institutions, and to pre-test the ECAP information. As panel members will be recruited from different locations, meetings will be held primarily online [29].

Below, we describe each of the following steps: 1) Mapping health and social care providers at regional and local level that currently provide ECAP information services to parents, 2) Assessing implementation facilitators and barriers from the mapped actors, 3) establishing a patient and public involvement group to advise the development and testing of a process to provide ECAP information to parents, 4), Developing exemplary ECAP information materials for distinct parent groups, 5) Initiating a phase of ECAP information provision, 6) Collecting and analyzing quantitative and qualitative implementation outcomes data.

### Assessment of implementation determinants

#### Part a: Mapping ECAP information providers

As outlined above, several on-site actors and institutions in health and social care may implicitly or explicitly provide ECAP information to parents. Therefore, it is necessary to understand who they are, and how providing ECAP information is part of their role. Mapping provides the basis for approaching health and social care providers for the subsequent steps of planning, realizing and evaluating the implementation.

### Data collection

Hannover in the north will serve as a model region for the mapping and will be complemented by two additional regions for comparison, Magdeburg in the east and Regensburg in the south, locations of other parts of the HELICAP consortium. The above-mentioned Public Health actors mapping by Hommes et al. (2021) provides the methodological basis for the subsequent steps [19]. Firstly, we will develop and agree on a preliminary set of search terms according to the Population-Intervention-Context-Outcome (PICO) logic (Table 1) [30]. The PICO scheme systematically addresses each of the items necessary to formulate a research question, i.e. to define a comprehensive search term for a bibliographic search. This will be done together with the parent panel and the scientific advisory board accompanying the project consortium (see Methods).

**Table 1:** Preliminary search terms according to the “Population-Intervention-Context-Outcome” scheme

Concept	Search term
#1: Population	Parents OR Family OR Children AND
#2: Intervention	Health information OR Education OR Parenting course OR Counselling OR Family center AND
#3: Context	Hannover OR [region 2] OR [region 3] AND
#4: Outcome	Allergy prevention OR Chronic disease prevention OR Health

Based on the final version, we will conduct a full digital search for regional and local institutions via Google, as this is the most common (digital) way for parents to search for health information [9]. For each full search term, two researchers will independently screen the first 100 results using inclusion and exclusion criteria. We will include those institutions/actors that provide:

- health and/or social care for children, including disease prevention, whether general or specific to allergies,
- information on child health to parents, either as part of the actor’s professional role of providing services to parents, or as part of a voluntary service, e.g. in the case of parents running a network, group, or initiative to advise other parents.

Correspondingly, we will exclude those that:

- are located outside the above specified regions,
- have no regular direct contact with parents,
- have no resources/working structures related to providing health information to parents,
- offer no information/consultation related to disease prevention.

We will then complete the findings using the snowball approach [31]. Firstly, websites of all included institutions will be screened for mentioned partners and collaborators who fulfil the inclusion criteria. Further, we will present the preliminary list to a conveniently selected group of regional and local public health and health care experts in the Hannover region as well as to the parents’ panel, for suggestions of further relevant actors. This step focusses on identifying parental health information providers/channels that may not be immediately identified through a formal search, but that nevertheless play a relevant role in communicating with parents about child health, e.g. “Neighbourhood Mothers”.

### Analysis

To structure the included sources, we will inductively assign each included actor to an overarching thematic category, based on their website’s description. According to the preferences for receiving ECAP information mentioned by parents in our previous work on this topic, categories could broadly relate to formal institutions such as a) health professionals specializing in child care and women’s health, b) family centers (childcare), c) community, civil and welfare services, and d) insurers; informal actors such as e) individuals and groups counselling/consulting other parents about child health (online and offline); f) associations/clubs run by volunteers offering exchange among parents; and g) voluntary workers.

#### Part b: Exploration of implementation barriers and facilitators

Once a map of ECAP information providers is available, the pre-implementation assessment dictates the exploration of determinants that may facilitate or hinder the provision of ECAP information to parents. According to the CFIR, these relate to an institution’s outer setting (e.g. external regulations that ease or hinder health information provision), inner setting (e.g. available resources) and characteristics of individuals (e.g. feeling capable of providing health information) within an institution. Hence, a qualitative approach is required to gain an in-depth understanding.

### Participants

We aim to conduct semi-structured interviews with health and social care providers from each mapping category, to cover the diversity of perspectives. Using the retrieved contacts from each included actor, we will approach them one at a time until *n* = 2 respondents from each category have agreed to participate (ca. *n* = 10–15 interviews in total). Contacts will be established, firstly, via email and, subsequently, by phone. We will follow a recruitment approach that takes the perspective of potential study participants [32]. Accordingly, we will for instance invite umbrella organizations of the local health and social care providers to assist the recruitment process, to increase awareness of and trust in the study.

### Data collection

Interview questions will be derived from the CFIR determinants, with a focus on aspects relevant prior to implementation, e.g. “relative priority”: “how important is it for you to provide ECAP information in your institution?”; “incentive systems”: “what kind of incentive would be necessary for you and for your clients to consider ECAP counselling/information?” The CFIR interview guide tool serves to draft a first set of questions, which will be reviewed and finalized by consulting experts from public health and implementation science who are associated with this study. The interviews should last 30–45 min and will be conducted according to the interviewees’ preference either via telephone, video call, or on-site. The project staff has experience in collecting qualitative data from several previous projects, e.g. [33].

### Data analysis

The interview transcripts will be imported into MAXQDA for qualitative content analysis according to Kuckartz [34]: In phase 1, two researchers will analyse *n* = 3 transcripts together using the CFIR codebook template and will adapt the preliminary list of codes according to the interview contents. In phase 2, two researchers will independently code the transcripts in full and identify any unclear text passages. These, as well as conflicts regarding coding, will be discussed and resolved in bilateral meetings between the researchers, in order to reach an inter-coder agreement. In phase 3, we will produce qualitative summaries for each CFIR subcategory included in the interviews and rate the determinants as barriers or facilitators using ratings described in [22].

### Data Integrity

Several measures will contribute to improving the research rigour for the qualitative phase of this study, i.e. the interviews: the use of a multi-coder approach and the ongoing comparison of the results will enhance the credibility of the analysis; the consistent application of the research methodology will ensure dependability; the review and discussion of the decisions that will be made during the research process in bilateral meetings will help to increase the confirmability. In addition, we will apply member checking for the coded text passages, meaning the interviewees will be asked to provide feedback on the analysis. We will initially do this for 20% of the analysed material to ensure feasibility, and ask for feedback on the remaining materials if this appears necessary.

## Implementation planning and realization

### Intervention design / implementation materials

To design information materials to be given to parents, we will create one basic ECAP brochure to address parents’ need for a general understanding of allergy prevention. The final format will be decided based on input from the parents’ panel (see Study Design). While there are various, especially digital ECAP information sources, e.g. from the German Allergy Information Service, a readily available resource does not currently exist. In terms of ECAP content, we will use the most recent version of the German allergy prevention guideline (2022) as a basis [35]. We will translate the 21 statements into short ‘memo sentences’, i.e. take-home messages parents may remember more easily. For this, it will be crucial to encourage parents’ ECAP behaviour by providing practical recommendations for daily care for the child. Attention will be given to formulate applicable recommendations while avoiding ‘prescriptions’: in our previous interviews, parents specifically asked for practical recommendations they could use during daily care for the child [7], a finding that has been described in other studies, too (e.g., [36, 37]). Then, a draft version will be reviewed by our parents’ panel. The project staff is experienced in translating scientific allergy evidence into user-centered formats [38].

Since we aim to test the provision of ECAP information, we will incorporate preferences for (additional) information formats – discussed during the pre-implementation interviews – for instance by transferring the contents of the basic information set into a short lecture/presentation to be delivered during on-site parental information events. This process will be informed by two meetings with the parent panel, one for initial agreement of intended formats, one for discussing first drafts. The aim is to produce three distinct ECAP information formats, from which those that provide the information can choose during the implementation phase. These could, for example, relate to visual summaries (e.g. display stands, posters for on-site display), oral conversation guidelines for personal counselling of parents during appointments, ECAP “to-go” (short notes and/or QR-codes that link to a video or podcast), or informational/educational giveaways (e.g. an overview of the most relevant digital child health information providers combined with an ECAP postcard). The project team’s research department is experienced in creating target-group–specific health information using distinct didactic formats (e.g. [39]) and can resort locally to professional media assistance.

### Implementation

#### Participants

When the materials have been prepared, we will approach all health and social care providers identified in the mapping to establish their interest in participation. We will first contact each in writing (e-mail or postal mail), and aim to increase the response rate by personal follow-ups, continuing until *n* = 5 institutions from each category built during the mapping are included (ca. *n* = 5–8 formal institutional/professional settings, ca. *n* = 3–5 informal, voluntary actors). Those with an initial interest will be asked a short set of questions (availability, opportunities for applying a specific ECAP information format; contacts for communication with the project team) to plan their participation.

#### Implementation phase

Based on the analysis of implementation barriers and facilitators and according to the IRLM, we will agree on up to three “implementation strategies” according to Waltz et al. (2019) – e.g. obtaining and providing regular feedback [24] – to be applied during the implementation, and elaborate the (technical) details of each strategy, e.g. when and how to provide feedback. The set of strategies will be offered to a random sample of half of the participating institutions to observe whether it affects the implementation outcomes (see below). For the actual implementation phase, each participating institution will be asked to approach parents during a period of up to 6 months. Table 2 describes core elements of the intervention according to the framework by Presseau et al. [40], i.e. the provision of ECAP information to parents.

**Table 2:** Implementation intervention framework

Domain	Definition
Action	Provision of ECAP information
Actor	health- and social care providers
Context	health- or social care facility
Target	parents of children (aged ≤ 3) with and without a specific interest in ECAP
Time	before, during or after parents interact with a health- and social care provider

To account for potentially distinct health information needs and preferences by parents with a direct, recent migrant background, we will adapt and extend the implementation planning and conduct for Arabic-speaking migrants. We will focus on this exemplary subgroup as it represents a significant proportion of the recent migrant population in Germany, i.e. about 1,5–2 million people are from Arabic-speaking countries [41]. In addition, to make use of the findings on this group from a previous study [42], we will first translate the distinct information formats. Further adjustments beyond language will be made when the pre-implementation assessment and planning reveals a need for this. Then, we will invite the previous interview partners to participate in the implementation process with a focus on approaching Arabic-speaking parents. Additional actors will be purposively selected upon necessity. Identical pre- and post-implementation interviews and survey questions will be applied to integrate the findings into the subsequent evaluation of implementation outcomes.

## Evaluation of implementation outcomes

The evaluation aims to explore how ECAP information providers perceive the implementation process (implementation outcomes) and how the recipients of information are affected (parents: consumer outcomes). To do so, we will apply implementation- and consumer-related outcomes defined by Proctor et al.’s Implementation Outcomes Framework [25], which is part of the IRLM.

### Data collection

We will develop a) a semi-structured interview guideline for qualitative interviews with information providers and b) a short, descriptive survey for parents. Regarding the interviews, questions will be formulated to explore acceptability, adoption, feasibility, fidelity (adherence), penetration (reach) and (expected) sustainability. These implementation outcomes relate to the CFIR domains of outer setting, inner setting and intervention characteristics [26], i.e. questions will be domain-specific (see Table 3).

**Table 3:** Exemplary and preliminary implementation evaluation questions

Item	Information providers	Information receivers
Implementation outcomes		
Acceptability and appropriateness	What were your experiences with the six-month test phase? How did it work out?	
Adoption	Did you have sufficient resources for this implementation process?	
Feasibility	Which kind of feedback have you received from those you gave the ECAP information to?	
Fidelity	To what extent did you adhere to the plan/process we agreed on?	
Penetration (reach)	How many parents did you reach?	
Sustainability	Would you consider sticking to this way of providing ECAP information from now on?	
User outcomes		
Satisfaction		Did the ECAP information you received provide an added value to you compared to how you learnt about ECAP before?
Comprehension		How understandable was the ECAP information?
Effectiveness		How likely is it for you to apply what you learnt about ECAP in daily life situations as a parent?

Regarding the survey, questions will be formulated to assess parents’ satisfaction with and comprehension of the kind of ECAP information they received, how they perceived the information provision process, and how effective the provision was. These outcomes primarily relate to the CFIR domains “intervention characteristics”, “characteristics of individuals” and “inner setting”, e.g.: “Comprehension (intervention characteristics)”: “How understandable did you find the ECAP information?” Both drafts will be discussed with the parents’ panel and scientific advisory board and be pretested (*n* = 3 interviews, *n* = 10 surveys).

Interviews with information providers: to evaluate institutions’ perspectives on the implementation, we will invite each actor involved in this process to participate in a 30-min on-site, digital or telephone interview. As those participating in the implementation process will have expressed their interest and willingness to do so, we assume that most will also agree to participate in the evaluation, and we will mention this important step during initial contact (step 3). Assuming that at least five formal and informal implementation settings can be distinguished (see step 2) from which five actors implement the ECAP information, respectively, we aim to conduct *n* = 25 interviews. The transcripts will be analysed separately using the same two-researcher approach as described for the analysis of pre-implementation interviews (see Part b: Data Analysis), according to ECAP information providers who were supported by implementation strategies and those that were not.

Survey for parents: we will link to a short standardized, electronic survey (SoSci Survey) in the provided ECAP information, as this seems more feasible for receiving parents’ feedback and likely to increases the response rate. To distribute the survey, a website link and QR-code will be attached to each ECAP information item provided to parents. In addition, each information provider will be asked to point parents at the evaluation. As we aim to include ca. *n* = 25 institutions/actors, these may on average reach *n* = 50 parents within 6 months, resulting in a total of ca. *n* = 1,250 parents (estimated response rate: 20%, [43]).

### Data analysis

The qualitative analysis of interview data is the same as that of the pre-implementation interviews (see above). Regarding the quantitative analysis of the survey data, data collected in SoSci survey will be exported to SPSS and surveys will be included for analysis when more than 80% of questions have been answered. Firstly, we will apply descriptive statistics by summarizing frequencies for each survey item, for example regarding the satisfaction expressed by parents with each of the distinct ECAP information formats. Secondly, bivariate analyses (crosstabs, chi-square test) will be used to explore relations between independent and dependent variables. For example, we will look at whether a parent’s educational status correlates with the use of the ECAP information they received, or if parents’ age impacts their preference for a specific type of ECAP information. Thirdly, ordinal logistic regression analysis will be used to identify potential predictors of ECAP-associated dependent variables, for example the utilization of information. To do so, we will include sociodemographic independent variables (e.g. completion of secondary school, A-levels, high school allergy risks of the child) in the model, i.e. survey. Lastly, we aim at one (mid-term) follow-up analysis, specifically to explore to what extent the recommendations are applied by parents over a longer period. Accordingly, we will follow-up those parents who agreed to be re-contacted six months after initial survey completion, and analyse the data using the methods described above. However, this follow-up will be understood as an additional element of the investigation, given that the focus is on understanding the implementation process, rather than mid/long-term individual effects.

## Discussion

Previous research on health information for parents has revealed that sources of information vary widely in content and quality [17], that information users have low awareness of where evidence-based information can be obtained and what the ‘right’ recommendations are exactly [44], and that there is a range of potential hurdles when communicating respective advice to target groups [45]. More generally, public health implementation research, for instance regarding the landscape of available institutions and organizations to support target groups in making good health-related decisions, appears to be scarce in Germany. Here, research has only recently begun to develop a more systematic understanding of which of Public Health services are being offered by which actors and to what extent [19]. One possible reason for this is that the focus often tends to be on developing and testing intervention studies, rather than going beyond this to implement sustainable processes for transferring scientific insights to patients and the public. As a consequence, as described above, the Public Health information landscape has been previously criticized as still not being fully developed [12]. This is particularly true regarding individuals and communities who either self-report problematic levels of health literacy, or who are challenged by not being confident about where to find information and advice for other reasons, e.g. when having recently migrated to a country that has a different type of health system compared to their country of origin [46]. In our planned study, we focus on Arabic-speaking migrant parents as an example for a group for which addressing potentially differing health information needs and preferences as well as outcomes might be particularly relevant, This is, not least, due to the differences Arabic parent experiences across health care systems and the differences related to accessing and applying health information, for which challenges have been previously reported in similar contexts [47].

Therefore, our study will initiate a multi-stage implementation science process, from assessing the implementation context including relevant actors and likely facilitators and barriers, to planning and implementing the provision of ECAP information to parents by health and social care actors, to evaluating the outcomes from the perspective of those who conducted the intervention, as well as those who are meant to benefit from this. In terms of content and the relevant stakeholders involved in this project, our study builds on several previous insights gathered by our research group (HELICAP). The scientific expertise gathered so far will inform this study at several stages, for instance during the development of the ECAP information materials. Since our study will investigate how ECAP information provision can be done effectively and what likely barriers may occur, we will communicate the most relevant findings to those who counsel parents on a regular basis towards the end of the project. Specifically, we aim to provide guidelines for ECAP counselling to relevant health and social care providers, to improve ongoing ECAP information provision.

Since the planned implementation process relies on intensive efforts by, resources from and participation of several external stakeholders, it is carried out within a defined and narrow area/setting and is therefore not representative of the entire (ECAP) health information landscape. Further, since we plan to involve professional and non-professional target groups in this study at several stages, it will be important to ensure participants’ interest and motivation, particularly because health care providers, for instance, are very occupied by their regular daily working duties. Besides financial incentives, we will thus need to provide additional, non-material added values such as the above-mentioned guidelines.

## Conclusion

From a Public Health perspective, there needs to be a good understanding about how evidence from health research can be effectively communicated to the public, rather than merely focusing for example on designing and testing interventions. In other words, implementation research project are important in the research field outlined here. Previously, it has also become clear that the dissemination of health information needs to better target those who face more substantial difficulties when seeking advice, i.e. individuals/parents who recently migrated. In this endeavor, ECAP may constitute a good use case, as scientific evidence is constantly evolving, and the communication of information is hampered by low awareness of high quality sources.

## Data Availability

No datasets were generated or analysed during the current study.

## Abbreviations

ECAP: Early childhood allergy prevention
CFIR: Consolidated framework for implementation research
HELICAP: Health literacy in early childhood allergy prevention
HL: Health literacy
HP: Health professional
IRLM: Implementation research logic model

## Footnotes

1 “social care institutions” here meaning actors that provide non-health related services to families, e.g., family centres that offer child care for parents, and municipal facilities that help parents with questions related to parenthood.
